# Complex Study of Magnetization Reversal Mechanisms of FeNi/FeMn Bilayers Depending on Growth Conditions

**DOI:** 10.3390/nano12071178

**Published:** 2022-04-01

**Authors:** Christina Gritsenko, Vladimir Lepalovskij, Mikhail Volochaev, Vladimir Komanický, Aleksandr Gorkovenko, Hanna Pazniak, Maria Gazda, Nikolai Andreev, Valeria Rodionova

**Affiliations:** 1 Research and Education Center “Smart Materials and Biomedical Applications”, Immanuel Kant Baltic Federal University, Gaidara str., 6, 236041 Kaliningrad, Russia; andreevn.misa@gmail.com (N.A.); valeriarodionova@gmail.com (V.R.); 2 Solid State Magnetism Department, Institute of Natural Sciences and Mathematics, Ural Federal University, 620002 Yekaterinburg, Russia; vladimir.lepalovsky@urfu.ru (V.L.); a.n.gorkovenko@urfu.ru (A.G.); 3 Kirensky Institute of Physics, Federal Research Center KSC SB RAS, Akademgorodok 50/38, 660036 Krasnoyarsk, Russia; volochaev91@mail.ru; 4 Institute of Physics, Faculty of Science, Pavol Jozef Šafárik University, Park Angelinum 9, 040 01 Kosice, Slovakia; vladimir.komanicky@upjs.sk; 5 Faculty of Physics and Center for Nanointegration (CENIDE), University of Duisburg-Essen, 47057 Duisburg, Germany; hanna.pazniak@uni-due.de; 6 Faculty of Applied Physics and Mathematics, Gdansk University of Technology, Narutowicza 11/12, 80233 Gdansk, Poland; margazda@pg.edu.pl; 7 Materials Science and Metallurgy Shared Use Research and Development Center, National University of Science and Technology MISiS, 119049 Moscow, Russia

**Keywords:** exchange bias, exchange spring, AFM grain size, substrate temperature, hysteresis loop asymmetry, magnetization reversal

## Abstract

Magnetization reversal processes in the NiFe/FeMn exchange biased structures with various antiferromagnetic layer thicknesses (0–50 nm) and glass substrate temperatures (17–600 °C) during deposition were investigated in detail. Magnetic measurements were performed in the temperature range from 80 K up to 300 K. Hysteresis loop asymmetry was found at temperatures lower than 150 K for the samples with an antiferromagnetic layer thickness of more than 10 nm. The average grain size of FeMn was found to increase with the AFM layer increase, and to decrease with the substrate temperature increase. Hysteresis loop asymmetry was explained in terms of the exchange spring model in the antiferromagnetic layer.

## 1. Introduction

For thin film materials with exchange-coupled ferro- (FM) and antiferromagnetic (AFM) layers, below the Neel temperature and at the induced uniaxial anisotropy, magnetic hysteresis loops are shifted. This phenomenon is called the exchange bias and is widely used in modern spintronics, magnetic recording, and magnetic sensorics, for example, in giant magnetoresistance (GMR)-based or giant magnetoimpedance (GMI)-based elements [1,2,3]. Depending on the application, the most important property of an exchange bias system can be either the effect value [4], or layer thickness [2,5], or hysteresis loop shape [5,6,7].

Experimental studies show that the magnetic properties of an exchange bias system are in the direct dependence on layer thickness, roughness, grain size, and deposition sequence [8]. The above characteristics of thin films prepared by magnetron sputtering can be varied by changing the pressure or substrate temperature in the chamber during deposition [9,10].

Ni_80_Fe_20_ alloy (Permalloy) is one of the most useful materials for exchange bias systems due to its high initial and maximum magnetic permeability, as well as corrosion resistance, when compared with other materials. For using it in GMR sensors, an optimal layer thickness is less than 20 nm. However, there are cases that require a thicker layer of Permalloy. For example, in realizing a magnetoimpedance (MI) effect, Ni_80_Fe_20_ layers are usually used with a thickness more than several tens of nanometers [2,11,12]. In such structures, exchange bias is used to provide asymmetry in MI response, wherein an exchange bias value and magnetization reversal behavior define the functional characteristics of exchange bias structures for their usage in applications such as GMI sensors. So, it is important to tailor magnetic properties of NiFe-based exchange bias structures with NiFe layer thicknesses of more than 10 nm.

Another important issue is the study of an AFM layer thickness influence on the exchange bias effect. For each particular structure, this influence will be unique, but the general tendency is an increase in the effect value with increasing AFM layer thickness from a few to tens of nanometers [13,14,15]. In this work, the Fe_50_Mn_50_ alloy was chosen as an antiferromagnetic material, which is commonly used in the mentioned industrial applications. Although it has poor corrosion resistance, it does not lead to a high enlargement of a pinning FM layer coercivity [16]; in addition, it is less expensive in comparison with other used AFM materials such as IrMn and PtMn [17]. 

Investigations of exchange bias, coercivity, and magnetoresistivity in FeNi/FeMn structures were performed recently, and dependences of these properties on the glass substrate temperature and annealing temperature were determined [3,18]. Experimental studies show that, in some cases, in FeNi/FeMn structures, the magnetization reversal process exhibits a hysteresis loop asymmetry [5,19]. In general, the two following features can cause this effect. The first one is a noncollinearity of uniaxial and unidirectional anisotropies in an FM/AFM system [20,21]. If the noncollinearity is presented, a hysteresis loop asymmetry is more manifested when the FM/AFM interfacial exchange coupling is stronger and FM anisotropy is larger. The second reason for the hysteresis loop asymmetry is structural imperfections at the FM/AFM interface (e.g., roughness) [19,22] that can give rise to the formation of exchange springs and planar domain walls in the AFM layer [19,22,23,24]. Recent studies show that the formation of exchange springs can be controlled by the temperature [19]. Particularly, a decrease in the temperature can promote exchange springs occurrence due to a weakening of thermal fluctuations and the enhanced ability of a domain wall to overcome the interface imperfections-induced energy barriers. However, it is not clear how the temperature conditions of a film growth and AFM-layer thickness affect this mechanism.

Accordingly, this work is aimed at a better understanding of a relationship between structural and magnetic properties of NiFe/FeMn thin films with thick layers up to 50 nm, depending on the antiferromagnetic layer thickness and substrate temperature. The study is also dedicated to a deeper understanding of the temperature dependence of magnetization reversal mechanisms in the structures.

## 2. Materials and Methods

The samples of Ta/NiFe/FeMn structures were prepared on Corning glass substrates using an ATC ORION-8 (AJA INTERNATIONAL) magnetron sputtering system in RF and DC mode. The pressure of the Ar atmosphere was 1.6 mTorr. The buffer Ta layer of 5 nm was preliminarily deposited on the substrate for better quality of other layer growth [3]. The compositions of ferro- and antiferromagnetic layers were Ni_81_Fe_19_ and Fe_50_Mn_50_, respectively. During the deposition, an in-plane magnetic field of 250 Oe was used to create uniaxial magnetic anisotropy in the samples. The thickness of the films was measured with a Dektak 150 stylus profilometer and the sputtering rate of NiFe, FeMn, and Ta was 1.2, 0.9, and 0.3 Å/s.

**Figure 1:**
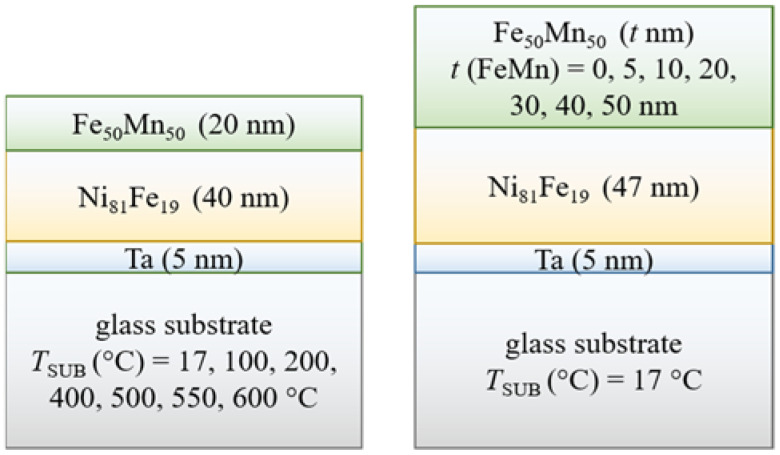
Schematic representation of the samples.

We have prepared two series of samples depicted in Figure 1. The first series of samples were glass/Ta (5 nm)/NiFe (40 nm)/FeMn (20 nm) structures with temperatures of the substrate during deposition of 17, 100, 200, 400, 500, 550, and 600 °C. The second series of glass/Ta (5 nm)/ NiFe (47 nm)/FeMn (t (FeMn) nm) structures with the AF-layer thicknesses t (FeMn) = 0, 5, 10, 20, 30, 40, and 50 nm was prepared at room temperature (17 °C).

Structural properties of the samples were carried out by transmission electron microscopy (TEM) using a Hitachi HT7700 at 100 kV accelerating voltage. Planar-view samples were prepared using a Hitachi FB 2100 (FIB) single-beam focused ion beam system. Powder X-ray diffraction (XRD) analysis was performed using the Phillips X’Pert Pro MPD with CuKα1 and CuKα2 radiation. The measurements were carried out in a 2*θ* range of 10–120° (0.02° step, 12 s per step) with 40 kV and 40 mA tube settings. Magnetic properties of all samples were investigated in the temperature range 80–300 K using the vibrating sample magnetometer (VSM) by Lake Shore 7400. In-plane hysteresis loops were obtained along the easy and hard magnetization axis.

## 3. Results

**Figure 2:**
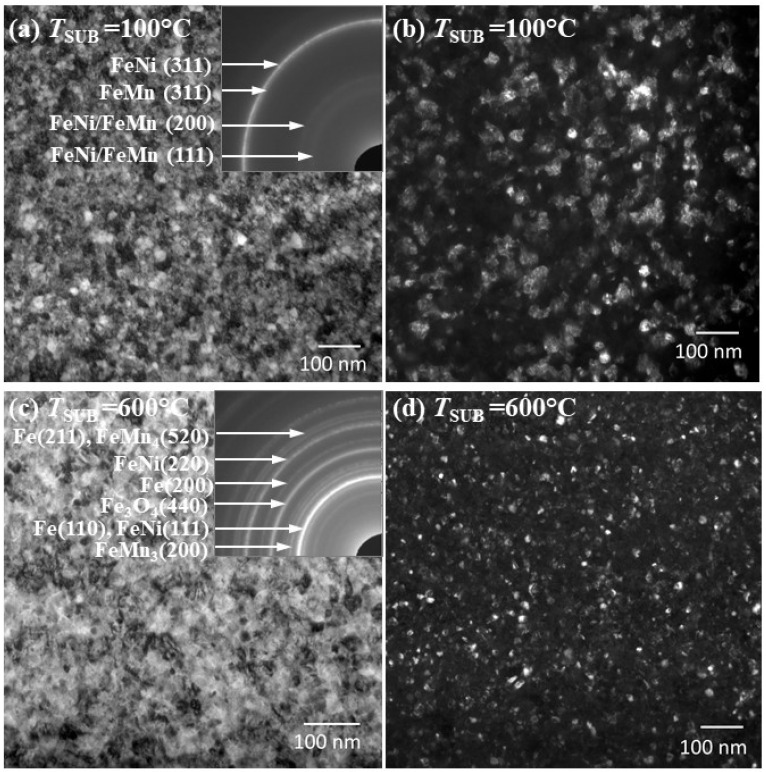
Planar TEM images and SAED patterns (insets in (**a**,**c**)) for thin films NiFe (40 nm)/FeMn (20 nm) deposited at: (**a**,**b**) 100 °C; (**c**,**d**) 600 °C of the substrate temperature.

The Selected Area Electron Diffraction (SAED) and TEM techniques were used to study the influence of the substrate temperature on the film structure. It was revealed that the growth of the AFM layer at the substrate temperature of 100 °C (Figure 2a,b) is formatted with the FeMn phase. At 600 °C (Figure 2b,c), there is a set of phases beyond the FeMn one: FeMn_3_, FeMn_4_, pure Fe phase, and Fe_3_O_4_ oxide phase. Apparently, at the substrate temperatures higher than 100 °C, the migration of atoms during deposition leads to the formation of these phases. This can happen because Mn atoms are known to have a relatively good diffusion mobility at a thermal treatment [25,26], as well as the FeMn layer having an increased oxidation ability [27]. Formation of the additional phases may reduce the amount of the antiferromagnetic FeMn phase that can lead to an exchange bias decrease as the temperature increases [18]. From the dark-field images (Figure 2b,d), it can be observed that an average grain size at the substrate temperature of 100 °C is larger than at 600 °C.

**Figure 3:**
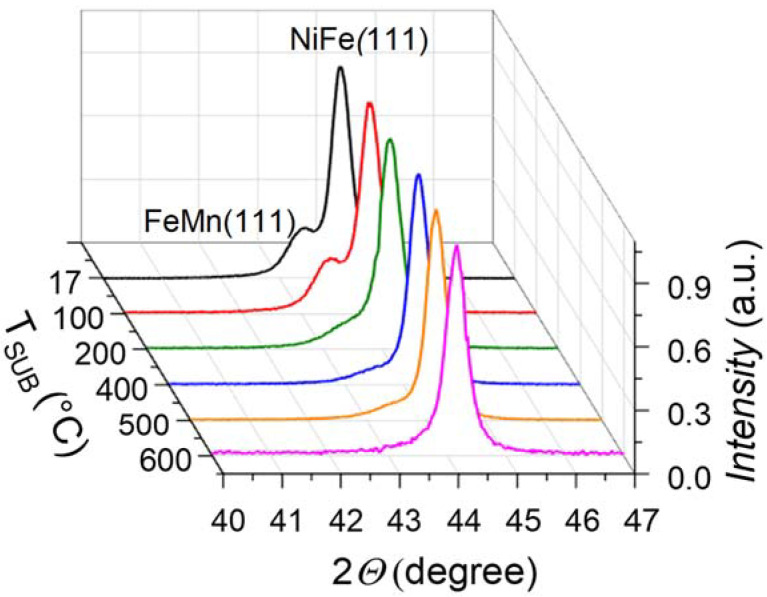
XRD patterns of NiFe (40 nm)/FeMn (20 nm) thin film structures deposited at different substrate temperatures (*T*_SUB_ (°C)).

XRD analysis was used to find out whether FeNi and FeMn layer grain size changes with the increasing substrate temperature. Figure 3 shows diffractograms of the films deposited at substrate temperatures of 17, 100, 200, 400, 500, and 600 °C. The XRD patterns show peaks corresponding to the FeMn and NiFe layers to have the fcc structure with (111) orientation that is usually observed in such structures [28,29,30,31]. Moreover, one can see that the peak intensity of the FeMn layer decreases with the increasing substrate temperature, while the width of the FeMn peaks increases with the increasing *T*_SUB_. The average grain size *d* of the layers was calculated using the Scherrer equation *d* = kλ/ Lcos*θ* [32], where k (0.89) is the Scherrer constant, λ is the X-ray wavelength, L is the relative value of the full width at half maximum (FWHM) of a peak, and *θ* is the Bragg angle.

The grain size *d* (FeMn) of FeMn (111) phase as a function of the substrate temperature during deposition is presented in Table 1.

For the FeMn layer, the average grain size is 12 ± 1 nm at the substrate temperatures of 17 °C and 100 °C. The average grain size of the FeMn layer decreased down to 7 ± 1 nm as the substrate temperature is increased from 200 °C to 500 °C. There is no visible peak of the FeMn phase in the XRD pattern at the substrate temperature of 600 °C. This means probably that the FeMn layer has too small average grain size. The observed tendency can be due to the found changes of the AFM layer with the increasing temperature, described above. Namely, it is possible that the occurrence of the Fe, FeMn_3_, and FeMn_4_ phases discourages an increase in the FeMn grain size. 

For the NiFe layer, the average grain size is 21 ± 1 nm and does not show any changes as a function of substrate temperature. The obtained values of grain size for both layers are consistent with previously obtained ones for similar structures [29,31].

Thus, based on the above diffraction studies, we can suppose that with an increase of the substrate temperature, an amount of the pure FeMn phase decreases, as well as its grain size decreases that is accompanied with an increase in the pure Fe phase. 

**Figure 4:**
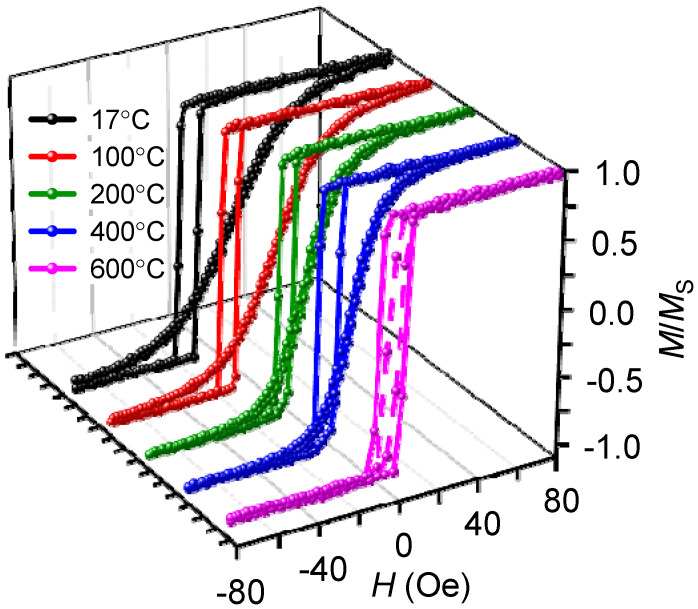
Hysteresis loops, measured at room temperature for the samples NiFe (40 nm)/FeMn (20 nm) with different temperatures of substrate during deposition, along a sample easy axis and hard axis.

For the structure of Ta (5 nm)/NiFe (40 nm)/FeMn (20 nm) deposited at different substrate temperatures, hysteresis loops along the easy axis have a rectangular shape (Figure 4), i.e., the magnetization reversal goes by a sharp domain wall propagation, while along the hard axis it goes by a gradual rotation of the magnetization. This behavior is typical for the NiFe/FeMn systems [33]. The coercivity along the hard axis is about zero for all samples except for the film deposited at the substrate temperature of 600 °C. With increasing substrate temperature, the slope of magnetization curves along the hard axis decreases. Thus, the last two findings indicate the weakening of the uniaxial anisotropy in the samples with the increasing substrate temperature.

**Figure 5:**
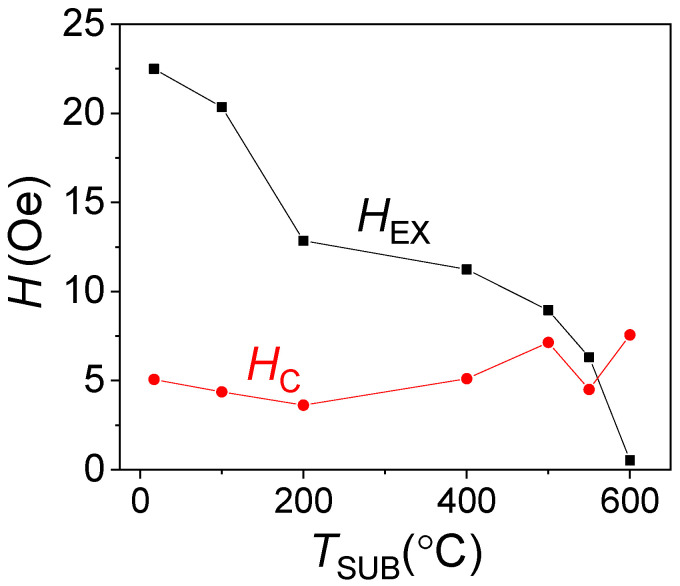
Exchange bias and coercivity dependence on the substrate temperature in NiFe (40 nm)/FeMn (20 nm) structures.

Dependences of the easy axis coercivity *H*_C_ (*T*_SUB_) and the exchange bias *H*_EX_ (*T*_SUB_) on the substrate temperature are shown in Figure 5. The maximal exchange bias of 23 Oe is achieved for the sample with the substrate kept at room temperature during deposition. With the increase in the substrate temperature, the exchange bias decreases inconsistently, meaning there is a large decline between *T*_SUB_ of 100 and 200 °C, and a small decline at the *T*_SUB_ range of 200–500 °C. We suppose it could be correlated with the observed change in the FeMn (111) phase grain size, indicated in Table 1, but this needs an additional study. At 600 °C, exchange bias almost vanishes. Thus, with the increasing substrate temperature the interfacial exchange energy weakens as it is in direct ratio with the exchange bias [8], wherein the increase in the substrate temperature does not change the magnetization reversal mechanism. In addition, coercivity of the samples does not change significantly as the substrate temperature increases, although the slight decrease up to 8 Oe is observed starting from 400 °C, which is related to a weakening of the AFM layer anisotropy. As the anisotropy weakens, AFM spins can follow FM spin at the interface during magnetization reversal, thus leading to the increase in the coercivity.

**Figure 6:**
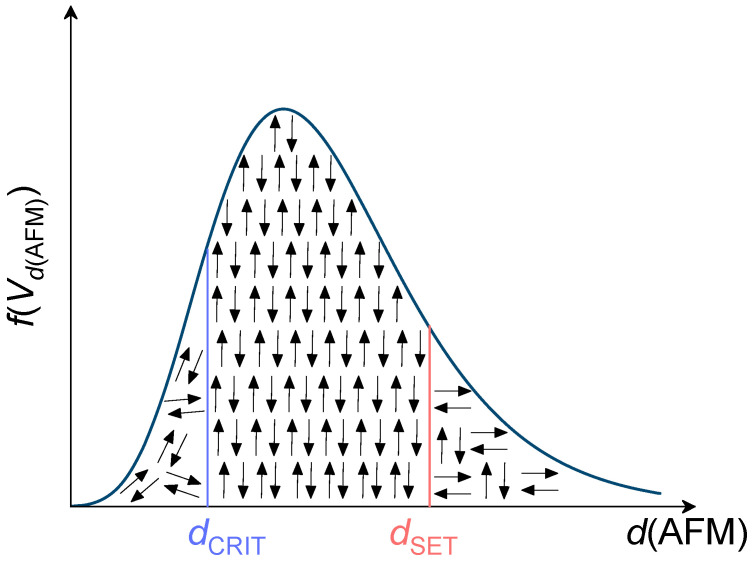
Schematic representation of an antiferromagnetic grain size (V*d*(AFM)) distribution after inducing of magnetic anisotropy.

Thus, it was found that as the substrate temperature increases, the FeMn grain size decreases as well as the exchange bias. This can be explained in terms of the thermal stability model [34,35], according to which at small grain sizeses below the critical *d*_CRIT_, an AFM layer contains a large fraction of thermally unstable grains (Figure 6). At grain sizes larger than some *d*_SET_, an AFM contains a portion of thermally stable grains but not set under applied magnetic field. Only at *d* in between these two critical values a larger fraction of AFM spins align with FM. In our case, at the given AFM layer thickness of 20 nm, the lower substrate temperature was during the film deposition, the smaller grain size was formed in the antiferromagnetic layer. This results in the formation of a larger fraction of thermally unstable grains in the FeMn layer, which leads to a decrease in the interfacial exchange coupling. A decrease in the exchange bias with decreasing FeMn grain size was also reported previously [36].

**Figure 7:**
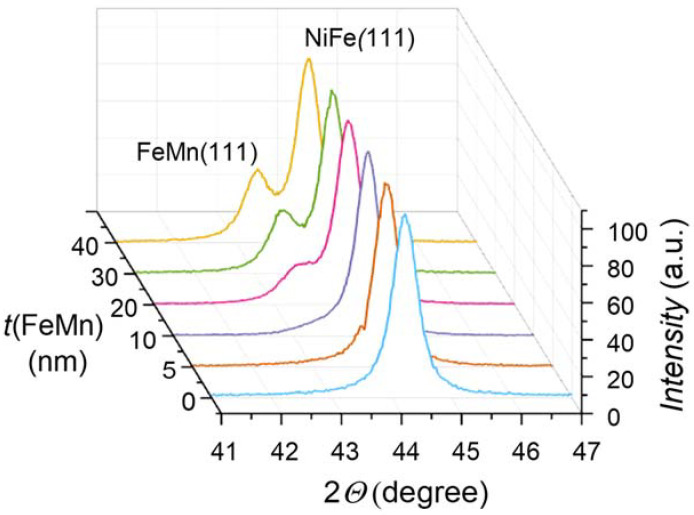
XRD patterns of NiFe (47 nm)/FeMn (t nm) thin films with various antiferromagnetic layer thickness deposited at room temperature.

NiFe (47 nm)/FeMn (*t* nm) structures were deposited at room temperature to study the effect of FeMn layer thickness on the sample properties. Figure 7 shows the diffractograms of the samples with 0, 5, 10, 20, 30, 40, and 50 nm thicknesses of the FeMn layer. Both layers, NiFe and FeMn, exhibit an fcc structure with (111) plane orientation.

The average grain size of NiFe layer is 18 ± 1 nm for all studied samples of this series that correlates with the structural features of NiFe layer observed by V. Vas’kovskiy et al. [3]. The estimated value of average grain size of the NiFe layer is less than in the previous series (NiFe (40 nm)/FeMn (20 nm)) due to the difference in layer thicknesses (47 nm in this series vs. 40 nm in the previous one). The grain size of the FeMn layer simultaneously increases with an increase in the film thickness, and the calculated values of the grain size are comparable with those previously obtained [37,38,39]. The grain size *d* (FeMn) of the FeMn phase as a function of the layer thickness is presented in Table 2.

**Figure 8:**
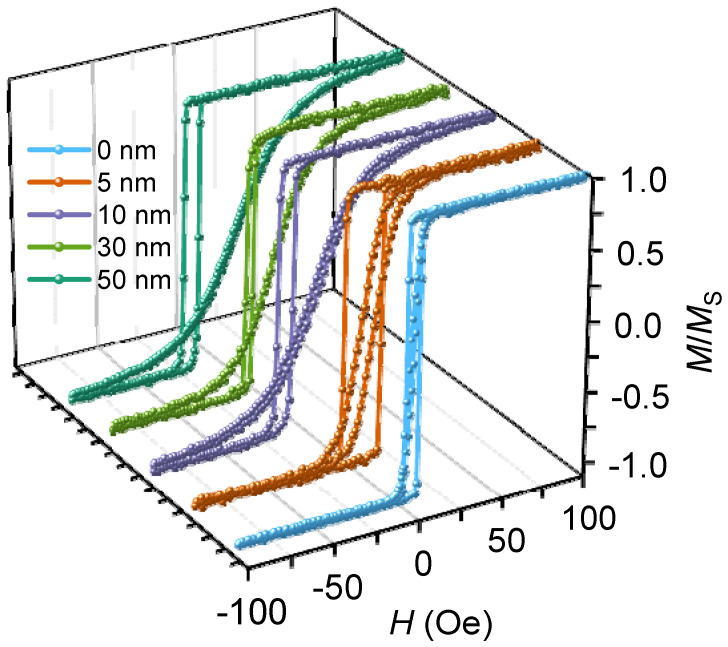
Hysteresis loops of NiFe (47 nm)/FeMn (t nm) thin-film structures with different FeMn layer thicknesses, along a sample easy axis and hard axis.

Hysteresis loops for the samples with 0, 5, 10, 30, and 50 nm of the AFM layer thickness are shown in Figure 8. All the loops along the easy axis have a rectangular shape that corresponds to the magnetization reversal by a domain wall propagation. Furthermore, along the hard axis, the slope of the hysteresis loops increases as the *t* (FeMn) increases. Therefore, the uniaxial magnetic anisotropy of the NiFe/FeMn system increases with the increase in the AFM layer thickness, wherein magnetization reversal mechanisms do not change. At 5 nm of the FeMn layer thickness, the coercivity along the hard axis is enhanced up to 5 Oe, and the exchange bias is only 2 Oe. This means that during magnetization reversal the interfacial AFM layer spins follow the FM layer spins under the torque [23]. In this case, anisotropy energy of the antiferromagnetic layer is not enough for a high exchange bias.

**Figure 9:**
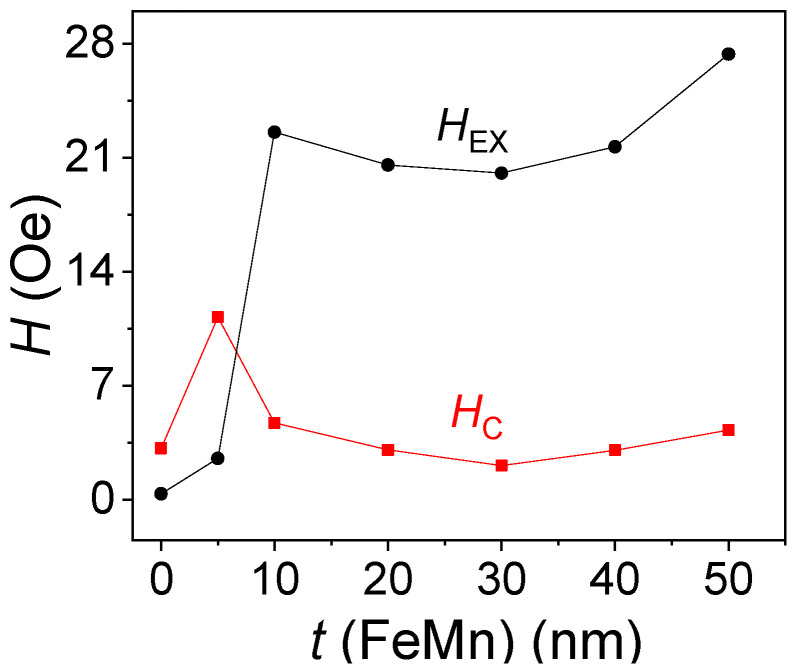
Dependences of the exchange bias and coercivity for easy axis on the antiferromagnetic layer thickness.

The dependences of exchange bias and coercivity on the FeMn layer thickness are presented in Figure 9 (for easy axis). *H*_EX_ is 2 Oe for the 5 nm-thick FeMn layer and 22 Oe for the 10 nm thick layer. A further increase in the antiferromagnetic layer thickness has no significant effect on the exchange bias. This behavior of the exchange bias vs. AFM layer thickness is typical for NiFe/FeMn bilayered systems [38]. After reaching a critical thickness, which in our case is 10 nm, the exchange bias does not noticeably increase. It can be explained via the observed AFM grain size change. Particularly, when the layer thickness is 5 nm, it contains small grains, and the AFM spins are thermally unstable, so they cannot contribute to a strong exchange bias. With the FeMn layer thickness increase up to 10 nm, a portion of unstable grains reduces, so the exchange bias correspondingly increases. As for the coercivity, it changes insignificantly in the range of 2–5 Oe, except at 5 nm for the AFM layer thickness, where *H*_C_ reaches the value of 11 Oe. This can happen due to the fact that unstable AFM spins are at the interface with FM ones, and upon magnetization reversal, affect the coercivity [8].

**Figure 10:**
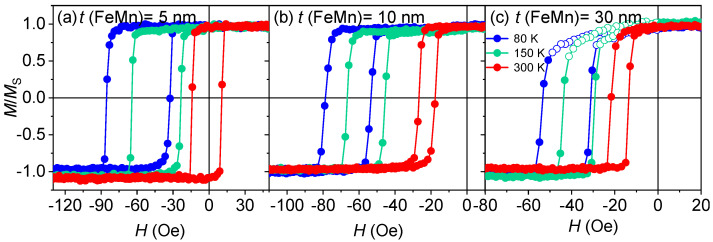
Hysteresis loops measured at 80 K, 150 K, and 300 K for the samples NiFe (47 nm)/FeMn (*t* nm) deposited at room temperature of the substrate. The thicknesses of the FeMn layer are: (**a**) 5 nm; (**b**) 10 nm; (**c**) 30 nm.

Magnetization reversal processes were studied using VSM at temperatures of 300 K, 150 K, and 80 K. Figure 10 shows hysteresis loops along the easy axis for the NiFe (47 nm)/FeMn (*t* nm) films deposited at room temperature. At 5 and 10 nm of the FeMn layer (Figure 10a,b), the hysteresis loop shape is rectangular, no asymmetry is observed, and the magnetization reversal process is irreversible [20,40] (i.e., domain wall propagation) and remains the same in the given temperature range. At *t* (FeMn) = 30 nm (Figure 10c) hysteresis loops are asymmetrical. At 80 K and 150 K, it is clearly seen that the hysteresis loops have regions with a more rounded shape (empty symbols in Figure 10c) which represents a reversible magnetization switching (i.e., gradual rotation of magnetic moments). 

**Figure 11:**
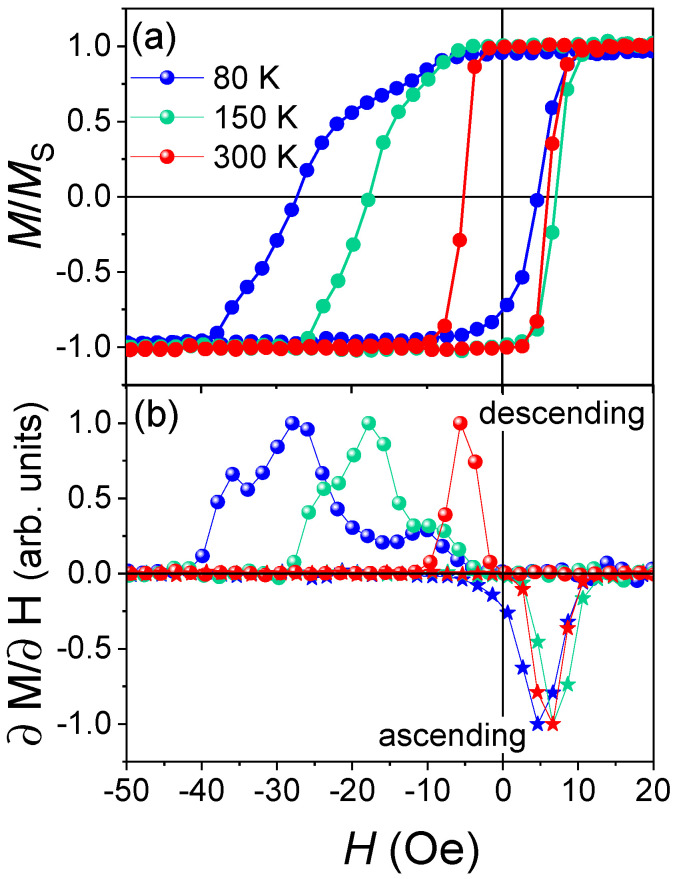
Hysteresis loops (**a**), measured at 80 K, 150 K, and 300 K, for the samples NiFe (40 nm)/FeMn (20 nm), deposited at the substrate temperature of 600 °C, and corresponding switching field distributions (**b**).

To distinguish whether the observed hysteresis loop asymmetry is just an effect of the grain size increase, or if it is caused also by the AFM layer thickness increase, we performed the study of magnetization reversal processes in the NiFe (40 nm)/FeMn (20 nm) sample deposited at the substrate temperature of 600 °C. This sample has an average grain size of the FeMn (111) phase less than 7 nm and the nominal layer thickness *t* (FeMn) = 20 nm. Figure 11 shows both the normalized hysteresis loops (Figure 11a) along the easy axis, and corresponding switching field distributions (SFDs) (Figure 11b) for the NiFe (40 nm)/FeMn (20 nm) films deposited at the substrate temperature of 600 °C. At 80 K, *H*_EX_ is 11 Oe and *H*_C_ is 16 Oe. The hysteresis loops at 80 K and 150 K are asymmetrical. At the descending magnetic field, the corresponding SFDs have a complex shape and are much more extended than at the ascending field.

Let us compare two samples: NiFe (47 nm)/FeMn (10 nm) deposited at room temperature substrate, and NiFe (40 nm)/FeMn (20 nm) deposited at *T*_SUB_ =600 °C. They have grain size of FeMn (111) phase 7 nm and less than 7 nm, respectively. For the first sample, the asymmetry of the hysteresis loop does not appear (Figure 10b), while for the second sample the hysteresis loop asymmetry is clearly observed (Figure 11a). Thus, the existence of the asymmetry for our samples is probably determined by the thickness of the antiferromagnetic layer, taking into account also the above-observed AFM layer phase separation. The observed type of the asymmetry can be attributed to the occurrence of an exchange spring in the AFM layer [41], because it is energetically more favorable to deform the antiferromagnet rather than to break the interfacial exchange coupling. In this case, the domain wall parallel to the interface in the AFM layer can contribute to the magnetization reversal [19,22,29,42,43]. In general, this effect is possible for promotion of a partial domain wall [23]. In our case, the observed hysteresis loop asymmetry is observed for the samples with AFM layer thicknesses above 10 nm. In this case, the appearance of the asymmetry with decreasing temperature can be explained by a decrease in grain thermal fluctuations, which leads to the stabilization of spins.

## 4. Conclusions

In this work, we study the influence of temperature of the glass substrate and the thickness of the antiferromagnetic layer on structural and magnetic properties of NiFe/FeMn exchange biased thin films. It was found that the grain size of the AFM layer decreases with the increase of substrate temperature from 17 °C up to 600 °C. In addition, the AFM grain size increases with increasing layer thickness. Exchange bias behavior vs. FeMn layer thickness, as well as vs. the substrate temperature, was explained in terms of the thermal stability model. In addition, the features of magnetization reversal of the samples were studied in the temperature range from 80 K up to room temperature. Asymmetry of the hysteresis loops at 80 K and 150 K was observed for the sample with the FeMn layer thickness more than 10 nm. This effect was explained by the appearance of the AFM domain walls parallel to the interface. In addition, the observed features can be useful for developing novel methods [44] for tuning the exchange spring in the AFM.

## Figures and Tables

**Table 1:** Average grain sizes of the FeMn (111) phase at various substrate temperatures during deposition.

*T*_SUB_ (°C)	*d* (FeMn) ± 1 (nm)
17, 100	12
200, 400, 500	7
600	<7

**Table 2:** Average grain sizes of the FeMn (111) phase at various thicknesses of the layer.

*t* (FeMn) (nm)	*d* (FeMn) ± 1 (nm)
5	<7
10	7
20	12
30, 40, 50	18

## Data Availability

Not applicable.
